# Sustained agreement rates in the longitudinal assessment of lupus patients to a Broad Consent for personal data and specimen usage in the RHINEVIT biobank

**DOI:** 10.3389/fmed.2023.1208006

**Published:** 2023-06-21

**Authors:** Jutta G. Richter, Tim Filla, Hasan Acar, Ellen Bleck, Anna Kernder, Christina Düsing, Stefan Vordenbäumen, Markus Schröder, Ralf Hansen, Jörg H. W. Distler, Matthias Schneider

**Affiliations:** ^1^Clinic for Rheumatology, University Hospital Düsseldorf, Medical Faculty of Heinrich-Heine-University, Düsseldorf, Germany; ^2^Hiller Research Center, University Hospital Düsseldorf, Medical Faculty of Heinrich-Heine-University, Düsseldorf, Germany; ^3^Department of Rheumatology, St. Elisabeth-Hospital Meerbusch-Lank, Meerbusch, Germany; ^4^Serrala Group GmbH, Berlin, Germany

**Keywords:** biobank, Broad Consent, systemic lupus erythematosus, quality management, RHINEVIT

## Abstract

**Background:**

Biobanks are essential structures for scientific research. The RHINEVIT biobank is used to recruit biomaterials from rheumatology patients in outpatient care and to conduct clinical research studies (e.g., cohort studies) and basic research. RHINEVIT established Broad Consents (BC) to allow extensive and relevant usage of data and biospecimens without the need for specific project restrictions. For quality assurance, we compared the consent rate of individual items of the BC versions in patients with systemic lupus erythematosus (SLE) in the longitudinal study.

**Methods:**

BCs were used for biomaterial donation. Informed consent data from RHINEVIT were analyzed. Due to the content restructuring of the BC items due to changes from the templates of the working group of the Medical Ethics Commissions in the Federal Republic of Germany and GDPR requirements, content mapping of the items was performed for the analysis.

**Results:**

From September 2015 to March 2022, 291 SLE outpatients donated biomaterials. In 119 patients, the BC was renewed at least once in a subsequent biomaterial donation. Three biomaterial donations were obtained from 21 patients and four from six patients using the respective BC. However, one consent was later revoked. Consent to the BC topics showed consistently high rates of agreement (range 97.5%−100%), with only some patients disagreeing with individual topics. This remained stable over time (median 526 days [Q1 400, Q3 844]). None of the patients disagreed with a certain topic in two consecutive visits.

**Conclusion:**

Modifications to the BC did not result in any relevant changes in the approval rates for SLE patients. RHINEVIT's BC is successfully used for the quality-assured handling of excellently annotated biomaterial. The long-term use of these highly valuable biospecimens for unrestricted research, also in an international context, remains assured.

## Introduction

Dedicated biobanks provide the opportunity to collect and store well-annotated human biomaterials for long periods of time. Their significance for modern medical research is still expanding, not only in “large, centralized high-quality biobanks” but also in those of clinical departments with specialized foci (1–3). Biomaterials in biobanks get annotated with medical and epidemiological data to increase their value. Thus, biobanks' infrastructures offer translational medical research the opportunity to identify, e.g., multi-omics-based biomarkers relevant for diagnosis, prognosis, therapy, and therapeutic response. Research goals include optimized prediction, risk assessment, and patient management for individualized diagnostic and therapeutic strategies (4). Apart from their complex infrastructures, biobank governance procedures that include ethical, legal, and societal implications (ELSI) and approaches play an important role in modern biobanking (5). Economic and social aspects of the biobanks, as well as those of individuals' care and current healthcare systems, have been taken into account (6).

Rheumatology biobanks may be used for the assessment of personalized treatment options (7). Since 2010, the Biobank “RHINEVIT” has been implemented at our clinic (8, 9). RHINEVIT is used for the recruitment of biomaterials from patients with inflammatory rheumatic diseases (IRDs) from our outpatient care at a university clinic for the purpose of conducting clinical and basic research not only at the local level but also within (inter-) national collaborations (8, 9). For example, the biobank was used for biomaterial collection within the CAPEA study (10, 11) and awareness campaigns (12). RHINEVIT provided biomaterial for genetic analyses (11, 13) and a study addressing nutrition in systemic lupus erythematosus (SLE) (14). The latter showed that dietary methyl donors may influence DNA methylation levels and disease activity (14).

Apart from study-by-study consent, RHINEVIT established a Broad Consent (BC) for biomaterial collection to assure modern, ethically acceptable biobanking (15). This approach provides sufficient flexibility to address research questions that are unknown at the time of biomaterial collection (15, 16). Nevertheless, the specific research questions are submitted to the local ethics committee for approval before the samples from the biobank are used for research projects.

Research on BC use in biobanking in IRDs is scarce, although there is an overarching need for a practice evaluation of BCs to improve their validity and applicability (16). Thus, for quality management reasons of the biobank and for its targeted further developments for use in IRDs in a real-world setting, it was considered necessary to explore the proportion of patients giving or withholding consent to BC topics like individuals' wish for feedback on incident findings.

## Methods

Since 2010, the Biobank RHINEVIT has used the certified biobanking software Genomatch University (manufacturer: Serrala Group GmbH, Berlin, Germany). The software complies with the necessary international standards, such as 21 CFR Part 11, Good Clinical Practice, and Good Laboratory Practice, and thus enables regulatory compliance for biobanking (17).

For the purpose of biomaterial collection in our routine outpatient clinic, BCs based on the templates of the “Arbeitskreis Medizinischer Ethikkommissionen in der Bundesrepublik Deutschland e.V.” (Medical Ethics Commissions in the Federal Republic of Germany) have been established as a governance procedure since 2014 (18). Owing to the adoption of the template of the “Arbeitskreis Medizinischer Ethikkommissionen in der Bundesrepublik Deutschland e.V.” and the EU General Data Protection Regulation (GDPR), our existing BC was always adjusted accordingly (16, 18, 19). Patients can approve or reject the detailed BC consent topics. Established withdrawal procedures were described in the patient's information. A revocation form is included.

When biomaterials were collected, the patient's information leaflet regarding the research scope of the biobank and its implemented procedures (e.g., accessing medical records for clinical and sociodemographic data), and consent forms were handed out as paper-based versions. Patients read the patient's information and considered their consent while waiting in the waiting room or in the outpatients' treatment room. Written informed consent was obtained by the treating rheumatologist or a member of the RHINEVIT biobank team. A signed copy was given to the patient to take the informed consent home. When a subsequent biomaterial collection was performed on an individual patient, the BC was renewed and entered into the software accordingly.

So far, the biobank stores only biomaterial taken in addition to blood for analyses within the scheduled routine outpatients' visits or in addition to non-biobank-exclusive research studies and does not store residual biospecimens (e.g., residual blood samples and tissues) (8, 9, 14, 20, 21). However, the BC already allows this. If patients were asked to donate biomaterial as part of a (clinical) research study (14, 20, 21), biomaterial donation to the biobank was possible regardless of their consent for that study.

An informed consent form was prepared for appropriate pseudonymization using patient's identifiers from the hospital information system and a pseudonym that was created for use within the biobanking software architecture (“Barcode 1”). This barcode 1 was also used for labeling the biomaterials to ensure that the information from the consent form and the biomaterials could be linked together appropriately.

Signed paper-based forms were entered manually by a trustee into the biobanking software via the provided trustee dashboard. It includes functionality to assign patients' identifying numbers (from the hospital information system) to the pseudonym (Barcode 1) and the current version of the informed consent. Annotating clinical parameters were derived from our web-based patient documentation system, DocuMed.rh (22). Patients' education was assessed in compliance with the German educational system. The Health Assessment Questionnaire (HAQ) index was calculated from the Hannover Functional Questionnaire (FFbH) (23). DocuMed.rh contains a greater number of patient-reported outcomes, such as, e.g., the systemic lupus activity questionnaire (SLAQ) and measures addressing health-related quality of life (24). The information could be linked to the biomaterial where necessary.

The use of BC in the context of the RHINEVIT biobank received its first positive ethical vote from the local ethics committee in 2014 (Number 4711) and was renewed with the updated BC versions. Studies with biomaterials were registered with the German Clinical Trials Register, where applicable.

For quality assurance purposes, data from three versions of informed BCs from the period of September 2015 until March 2022 were extracted from the biobank database. The consent options of the versions were mapped to each other to guarantee the comparability of items.

None of the BC items distinguished between genetic, serological, and/or clinical parameters that affect or derive from the biomaterials. However, patients were informed in the patient's information that genetic data or examinations might be performed.

As most patients with systemic lupus erythematosus (ICD-10-Code M32.^*^) were asked for their informed consent for RHINEVIT on a longitudinal basis in our outpatient clinic, we analyzed their responses to the Broad Consents.

### Statistical analyzes

Values were expressed as percentages and absolute numbers for discrete variables or as medians with a 25 and 75% quantile for continuous variables. For statistical analysis of multi-class variables, a chi-squared test was used. A *p*-value < 0.05 was considered significant. All statistical computations were conducted using R version 4.2.1. The data were analyzed anonymously.

## Results

Overall, 291 SLE outpatients consented to the various versions of the BC. For 249 patients, the biomaterial was collected as a voluntary add-on to another research project.

The patients' median age was 44 years. Most patients were women (86.3%). The median disease duration was 15 years. Further clinical characteristics of the cohort are presented in Table 1.

**Table 1:** Listing of socio-demographic and clinical data of *n* = 291 SLE patients [BC Broad Consent, Q1 first quartile, Q3 third quartile, SLEDAI Systemic Lupus Erythematosus Disease Activity Index, HAQ Health Assessment Questionnaire (Score calculated from the Funktionsfragebogen Hannover (FFbH))].

	**All**	**Consented to all BC items**	**Lack of consent on individual BC items**
*N*	291	286	5
Age, median [Q1, Q3]	44.0 [33.8, 53.0]	44.0 [33.0, 53.0]	40.5 [39.3, 48.5]
Sex = female, *n* (%)	251 (86.3)	246 (86.0)	5 (100.0)
Education, *n* (%)		
University entrance diploma	136 (47.4)	132 (46.8)	4 (80.0)
Another school diploma	143 (49.1)	143 (49.6)	0 (0.0)
No school diploma	8 (2.8)	7 (2.5)	1 (20.0)
Disease duration in years, median [Q1, Q3]	15.0 [8.0, 21.0]	15.0 [8.0, 21.0]	16.0 [7.0, 22.8]
Duration of care in our center in years, median [Q1, Q3]	10.5 [5.4, 15.5]	10.5 [5.3, 15.4]	15.1 [11.8, 15.7]
Co-consent to other studies performed at our center, *n* (%)	249 (85.6)	245 (85.7)	4 (80)
Ever Co-consent to other studies performed at our center, *n* (%)	278 (93.8)	273 (95.5)	5 (100)
SLEDAI score, median [Q1, Q3]	2.0 [0.0, 5.0]	2.0 [0.0, 5.0]	4.0 [3.0, 6.0]
FFbH score, median [Q1, Q3]	94.4 [77.8, 100.0]	94.4 [77.8, 100.0]	91.7 [43.1, 98.6]
HAQ score, median [Q1, Q3]	0.52 [0.36, 0.98]	0.52 [0.36, 0.98]	0.44 [0.36, 0.63]

In a total of 119 patients (median age 47.6 years [Q1 37.5, Q3 55.8], 66% female), consent was renewed for subsequent biomaterial collections at least once. Hereby, we identified 21 patients with three and six patients with four biomaterial collections, resulting in 443 Broad Consents. Only one consent was revoked for unknown reason(s).

Regarding the differences in clinical characteristics between patients who gave consent to all items and those who refused some items, we observed that most characteristics are similar in both groups. Only for the education level, a significant difference was identified, with those who refused at least one item having a higher level of education (a university degree) at a higher rate [80.0% (*n* = 4) vs. 46.8% (*n* = 132), *p*-value Fisher exact test: 0.04].

The detailed results of the consent rate for each item are shown in Table 2. Biomaterials' ownership was transferred to RHINEVIT by 98.9% (*n* = 438; Item 2). Of those who consented, 98.9% (*n* = 438) approved that the purposes of scientific-medical research their biomaterials and data are used for are not limited (Item 1), and 98.9% (*n* = 438) consented to the use of their biomaterials and data for medical research projects for an unlimited period, respectively, for 30 years (Item 9).

**Table 2:** Consent rates for different items of the Broad Consent (BC) and different biomaterial sampling dates.

**Item of the BCs**	**Consent rate (%)**				
	**Total**	**First consent** ***n*** = **291**	**Second consent** ***n*** = **119**	**Third consent** ***n*** = **27**	**Fourth consent** ***n*** = **6**
Item 1: the purpose of the research is not limited	98.9	99.3	97.5	100	100
Item 2: transfer of biomaterial ownership to biobank	98.9	99.3	97.5	100	100
Item 3: contact possible at a later timepoint	98.4	98.6	97.5	100	100
Item 4: contacting for further information/obtaining further biomaterials	99.8	99.7	100	100	100
Item 5: contact to obtain consent to link further medical data from other databases	100	100	100	100	100
Item 6: contacting for feedback on “health-related incident findings”	100	100	100	100	100
Item 7: contacting other physicians to obtain information about the course of the disease	100	100	100	100	100
Item 8: storage of personal data/e.g., transfer of clinical data from other systems and linkage with the biomaterials for research	98.9	99.3	97.5	100	100
Item 9: use of biomaterial for an unlimited period of time (since 2021 for the next 30 years)	98.9	99.3	97.5	100	100
Item 10: pseudonymised transfer of biomaterials to, e.g., universities/research institutions also in foreign countries	98.4	98.6	97.5	100	100

Similarly, 98.9% (*n* = 438) agreed that the biobank collects/extracts additional information on their health from their health records and merges these data with the biomaterials available to RHINEVIT in pseudonymized form for medical research projects (Item 8). Everyone agreed to obtain feedback concerning significant individual health issues (“health-related incident findings”) resulting from potential research on their biospecimen (Item 6).

In total, 98.4% (*n* = 436) of patients agreed that they might be contacted at a later point in time (Item 3), while 99.8% agreed that they might be contacted for further information and biomaterial extraction (Item 4).

Moreover, 98.4% (*n* = 436) agreed that their biomaterials and data may be transferred in pseudonymized form to universities, research institutions, and research companies, possibly also abroad (with probably lower data security regulations), for medical research purposes (Item 10).

All agreed to be re-contacted to obtain consent to link further medical data from other databases (Item 5) and to contact other physicians, other medical specialists, or hospitals to obtain information about the course of the disease (Item 7).

The median time difference between the first and second outpatient visit dates for biomaterial donation was 526 days [Q1 400, Q3 844]. The consent rate for the detailed items of the BCs was slightly lower on the second visit date but remained at a very high level, with at least 97.5% for each item. None of the patients disagreed on a certain topic on two consecutive visits.

## Discussion

To the best of our knowledge, our study is the first to focus on the individual BC in a rheumatology biobank embedded in routine care. The various aspects of our established RHINEVIT BCs that were and are based on the template of the Medical Ethics Commissions in the Federal Republic of Germany were well accepted by our predominantly female SLE patients, also over time with the changing BCs. This is in contrast to recent findings from a survey among participants in a large DNA biobank, where the authors conclude that biobank participants' preferences regarding sample use may change over time and consent stability cannot be taken for granted (25). They also deduce that this situation is a supporting reason for establishing a dynamic consent mechanism or an interface that allows patients to manage their participation in biomaterial donations (25). We have already identified the latter and are currently under software development, although only one patient withdrew informed consent.

Our biomaterial donors that consented to all BC items are of middle age, while those denying some items were a little younger (see Table 1). This is in line with previous studies reporting that the donation of biomaterials and clinical health information is higher in middle and older age (26). The difference in the mean HAQs between the two groups did not meet the minimal clinically important difference (MCID) and was thus regarded as neglectable. The low number of FDA/EMA-approved therapies for SLE may be a motivating factor for our patients to agree to biomaterial donation for biobanking and the associated research opportunities, as common motives for donation are prosocial reasons, e.g., the benefit of future patients (15, 26).

Biomaterial donation and the future of biobanking research also heavily rely on trust in biobank owners and the relationship with the researcher (26–28). Although we did not assess the reasons for biomaterial donation, and, in particular, patients' trust in us as their caring physicians and the Rhinevit biobank owners, directly from the patients, we used the duration of disease and duration of care in our clinic as proxies. Both revealed long durations, which might explain our consent rates. However, those with a lack of consent on individual items of the BC showed the longest duration of care in our center, questioning this argument. In addition, the cohort's high rate of co-consent to other studies run at our clinic might serve as a proxy for trust. The previously reported patients' preference for BCs over study-by-study consent models did not seem to play a relevant role (29).

Richter et al. recently reported from a German outpatient study (*n* = 650) that the belief that every citizen has a duty to contribute to the improvement of medical research is the strongest predictor of positive attitudes toward data donation (30). To our knowledge, unfortunately, there are no data on SLE patients' perspectives on this. Therefore, it is not possible to assess whether these beliefs play(ed) a role in our cohort, indicating a field of research. However, the strong positive attitude reported by German citizens in support of medical research might be taken as a “given prerequisite” (31).

Fortunately, in our analyses, no BC item was consistently disagreed with. Thus, no BC item was detected as an “imposable item.” Our few patients who disagreed with individual items had high levels of education. Education has been identified as a relevant factor for knowledge and acceptance of biobanks (1). As reported by Pacyna, patients may not have thought about the content and possible implications of their restriction the first time, but they thought about it more thoroughly when the topic was re-presented with the next BC (25).

Although our biomaterial donors do not routinely receive information on secondary findings according to the biobank's information leaflet, and although there are significant discussions among researchers and institutional review boards about how to deal with secondary and research findings (e.g., mechanisms and limited resources) (32, 33), all our patients were interested in these incidental findings. Patients' improved understanding of the administrative and financial burdens as well as the personal and moral implications for them personally (e.g., susceptibility to relevant yet unknown diseases) would possibly lead to different opinions (34). In our experience, close collaboration between biobank users and the respective ethics committee is of paramount importance. For instance, in the case of treatable pathological conditions that may be detected in the course of an initially unanticipated research project using biobank specimens, individual patients may have to be contacted to receive counseling from an ethical point of view (11).

However, that understanding and the resulting opinion require extensive health literacy and individual knowledge that cannot be taken for granted. Furthermore, contextual demographic, cultural, and sociopolitical factors need to be taken into account (34). (e)Health literacy is still limited, especially in rheumatology patients (35, 36), and knowledge about biobanking and biomaterial donation is usually similarly lacking, so educational processes would lead to enormous efforts in the biobank teams (32, 34, 37). This holds true also for rheumatology care in the United Kingdom, where patients' knowledge of biobanks was described as still limited even though a large nationwide biobank is in place for a large population-based prospective study (1, 38). Limited biobank funding may hinder the establishment of appropriate governance infrastructures, including patient and staff education and marketing (39).

Unfortunately, apart from publications naming biobanks (40–42), to date, no official numbers of SLE biobanks are available for Germany or Europe. The use of informed consent has not been studied systematically, and information on them is scarce within the published manuscripts. Therefore, these issues will be addressed by a survey from the European Reference Network, ReCONNET (43).

Recently, the RHINEVIT BC was updated, allowing the storage of additional skin biopsies. Further efforts to include other biomaterials, such as kidney biopsies, are ongoing. Since additional annotations of biomaterials from other data sources, such as laboratory tests from primary care physicians, orthopedic surgeons, or dermatologists, can be highly valuable for biomaterials in biobanks, the implementation of these further annotation possibilities for RHINEVIT will be sought in the future.

Our patients extensive support of research gives us high flexibility for performing our research with biobanked biomaterials. However, it is fundamental to implement and actively use policies on biomaterials and annotating data sharing that relate to ethical, legal, and societal issues (ELSI) and also meet the specifications of the institutional review boards (29). Typical documents covering the ELSI aspects on a European level are available from the “Biobanking and Biomolecular Resources Research Infrastructure—European Research Infrastructure Consortium” (BBMRI-ERIC) and the national nodes (44).

## Limitations

In this study, predominantly female patients with SLE were studied at a tertiary rheumatology center. Further studies involving more men and other IRDs are needed. The majority of patients were asked to donate additional biomaterial as co-consent for a clinical research study. In addition, we did not investigate why patients gave or withheld their consent and do not yet have a picture of patients' understanding of the contents of our BCs. RHINEVIT does not yet systematically assess if patients have an interest in results emerging from biobank activities. These issues need to be addressed in further research.

## Conclusions

The modifications made to the template text of the Working Group of the Medical Ethics Commissions in the Federal Republic of Germany and the resulting adaptations of the BC have not led to any relevant changes so far in the consent rates for SLE patients participating in RHINEVIT. Thus, our BCs have been and are successfully used for quality-assured handling of excellently annotated biomaterials from routine care. The study showed that there is a high willingness among our patients to consent to the (additional) collection of biomaterials and the use of their data collected within routine care for research, even in the context of a BC and without pre-specified research aims. Further research on patients' preferences that also assesses the practicability and acceptability of electronic infrastructures to support the contact of participants and dynamic consents in biobanks is warranted.

## Data availability statement

The data supporting the conclusions of this article are available on reasonable request by the authors.

## Ethics statement

The study was conducted under the approval of the Ethics Committee of Medical Faculty at Heinrich-Heine-University Duesseldorf for the biobank, local study number 4711. Written informed consent for participation was not required for this study in accordance with the national legislation and the institutional requirements.

## Author contributions

All authors were involved in the biobank project, made substantial contributions to the conception and design of the work, the acquisition, analysis, and interpretation of the work, developed the manuscript, and approved the final draft.
